# Internal Strangulation of the Intestine by Cicatrices

**Published:** 1876-01

**Authors:** 


INTERNAL STRANGULATION OF THE INTESTINE BY CICATRICES DUE TO AN HÆMATOCELE.—M. Antoine Magnin, Hospital Interne, reports the following case: A woman was admitted to the hospital suffering from obstinate constipation and abdominal pain, with tenderness on the left iliac fossa. The bowels had not moved for fifteen days. The patient had been delivered of a seven-months' child one year before. Two months after the delivery her menses had returned, but with only scanty discharge, and accompanied with prolonged pains in the abdomen. At the time of admission to the hospital the menses had not appeared for two months. During the succeeding days the pains continued and increased, and were associated with vermicular contractions of the intestines, which were distinctly visible externally. On making a vaginal examination the uterus was found to be fixed in an immovable position, and in the vaginal cul de sac a hard surface was felt, which extended somewhat to the left of the uterus. The symptoms grew more severe, the contractions became more painful, the intestines were tympanitic, and vomiting began. The vomited matters were at first simply alimentary, and afterwards fæcal. The patient gradually sank, and died in collapse about one month after her admission to the hospital. At the autopsy signs of a recent peritonitis were found in the pelvic cavity, with a slight serous effusion, but no fæcal matter. The large intestine was greatly distended, and filled with fæces. On a line with the posterior superior border of the uterus a circular strangulation was found, which had been produced by cicatrical bands, which united the whole of the posterior portion of the uterus to the rectum and parts adjoining. A short distance above the strangulation a perforation was found, through which, on using some little force, a portion of the contents of the bowel could be forced into the peritoneal cavity.—*Lyon Medical*, Sept. 19, 1875.—*Med. Record.*

